# Use of FDG-PET to guide dose prescription heterogeneity in stereotactic body radiation therapy for lung cancers with volumetric modulated arc therapy: a feasibility study

**DOI:** 10.1186/s13014-014-0300-9

**Published:** 2014-12-23

**Authors:** Bénédicte Henriques de Figueiredo, Mikael Antoine, Renaud Trouette, Philippe Lagarde, Adeline Petit, Frédéric Lamare, Mathieu Hatt, Philippe Fernandez

**Affiliations:** Department of Radiotherapy, Institut Bergonié, 229, cours de l’Argonne, F-33076 Bordeaux Cedex, France; Department of Radiotherapy, Hospital Haut-Lévêque, CHRU Bordeaux, Bordeaux, F-33076 France; Department of Nuclear Medicine, Hospital Pellegrin, CHRU Bordeaux, Bordeaux, F-33076 France; LaTIM INSERM, U1101 Brest, France

**Keywords:** Stereotactic body radiation therapy, Lung cancer, Positron emission tomography, Volumetric modulated arc therapy

## Abstract

**Background:**

The aim of this study was to assess if FDG-PET could guide dose prescription heterogeneity and decrease arbitrary location of hotspots in SBRT.

**Methods:**

For three patients with stage I lung cancer, a CT-simulation and a FDG-PET were registered to define respectively the PTV_CT_ and the biological target volume (BTV). Two plans involving volumetric modulated arc therapy (VMAT) and simultaneous integrated boost (SIB) were calculated. The first plan delivered 4 × 12 Gy within the PTV_CT_ and the second plan, with SIB, 4 × 12 Gy and 13.8 Gy (115% of the prescribed dose) within the PTV_CT_ and the BTV respectively. The Dmax-PTV_CT_ had to be inferior to 60 Gy (125% of the prescribed dose). Plans were evaluated through the D95%, D99% and Dmax-PTV_CT_, the D2 cm, the R50% and R100% and the dice similarity coefficient (DSC) between the isodose 115% and BTV. DSC allows verifying the location of the 115% isodose (ideal value = 1).

**Results:**

The mean PTV_CT_ and BTV were 36.7 (±12.5) and 6.5 (±2.2) cm^3^ respectively. Both plans led to similar target coverage, same doses to the OARs and equivalent fall-off of the dose outside the PTV_CT_. On the other hand, the location of hotspots, evaluated through the DSC, was improved for the SIB plans with a mean DSC of 0.31 and 0.45 for the first and the second plans respectively.

**Conclusions:**

Use of PET to decrease arbitrary location of hotspots is feasible with VMAT and SIB for lung cancer.

## Introduction

Stereotactic body radiation therapy (SBRT) is considered as a standard treatment for patients with inoperable early stage lung cancer and leads to local control rates over 90% [1-4]. Historically, SBRT is delivered using a large number of non-overlapping treatment beams or arcs to create a sharper dose gradient between the target volume and the surrounding normal tissues. These delivery techniques result in large dose heterogeneity with a frequent dose prescription specified at the 80% (or lower) isodose and hotspots within the planning target volume (PTV). The recent ROSEL guidelines about SBRT recommend the maximum PTV dose to be between 110% and 140% of the prescription dose [5]. However, the location of these hotspots within the PTV is uncertain and uncontrolled by conventional SBRT delivery techniques.

Recent developments including volumetric modulated arc therapy (VMAT) with one or multiple arcs and varying openings of the multi-leaf collimator (MLC), dose rates and gantry speed, have resulted in more homogeneous dose distributions in SBRT [6]. However, clinical results of SBRT have historically been obtained with overdoses ranging from 120 to 140% inside the PTV, which possibly contribute the success of SBRT. In addition to delivering more homogeneous treatments, if used with simultaneous integrated boost (SIB), VMAT can also create a dose heterogeneity and controlled hotspots inside the PTV.

For non-small cell lung cancers (NSCLC), positron emission tomography (PET) with [^18^F]-Fluorodeoxyglucose (FDG) is frequently used to optimize radiotherapy for advanced stages with a better distinction between tumour and atelectasis, and a better detection of involved nodes. PET is not used for SBRT planning because the definition of the gross tumor volume (GTV) on computed tomography (CT) imaging in NSCLC stage I cases is not difficult. However, several studies have shown that FDG-PET could detect the sites at risk of failure inside GTV [7,8]. These observations led us to use FDG-PET to guide hotspots and dose prescription heterogeneity for NSCLC SBRT with VMAT.

For this purpose, a feasibility study was conducted by analyzing the dosimetries of three patients. For each patient, two VMAT plans were performed and compared: the first plan delivered SBRT using only CT data, whereas the second plan delivered SBRT using CT and FDG-PET data to guide dose prescription heterogeneity.

## Material and methods

### Patient group

Three patients, enrolled in a clinical trial assessing the impact of 4D-FDG-PET in radiotherapy for lung cancer, were selected for this dosimetric study. The study was approved by the institutional review board of the universitary hospital and the French health authority and patients gave written informed consent to participate. All patients were male with a median age of 70 years [62–72] and peripheral T1N0M0 NSCLC (two adenocarcinomas and one squamous cell carcinoma). The tumour was located in the left inferior lobe with attachment to the chest wall for patient 1, in the middle lobe for patient 2, and in the left upper lobe for patient 3.

### Imaging

For CT simulation, patients were in supine position with the arms above the head using an armrest. Data were acquired with a 3-mm slice in free breathing. No four dimensional (4D) acquisition was performed because 4D CT scan was not implemented in our department at the date of this study. For FDG-PET/CT, a 4D acquisition was performed in radiotherapy treatment position, 50 min after intravenously injection of 3.7 MBq/Kg of [^18^F]-FDG, on a PET/CT integrated system (Discovery RX, General Electric Medical System®, Milwaukee, WI) using the Real-time Position Management (RPM) device (Varian®) and the “Motion Free” software (General Electric Medical System®). The respiratory cycle was rebinned in six phases.

### Target volumes definition

For the target volume definition, CT simulation and PET/CT were registered using a rigid algorithm. The GTV_CT_ was delineated manually on CT simulation using a parenchymal window (−1000 and +200 Hounsfield Units). Patients 1 and 3 presented a non-mobile lesion (located in the upper lobe or attached to the chest), therefore a 5 mm isotropic margin was added to GTV to create PTV_CT_. For patient 2, a 8 mm isotropic margin was added.

The Biological Target Volume (BTV) was defined on 4D-PET and corresponded to the sum of the six BTV delineated on the six respiratory-gated PET images. BTV was delineated on each PET image using the fuzzy locally adaptive Bayesian (FLAB) method (9), which is an automatic segmentation method. This statistic algorithm combined with a fuzzy measure is particularly adapted to noisy and blurry PET images, and has been validated on both simulated and clinical datasets for accuracy and robustness. Also, BTV included internal target volume (ITV) in its definition.

The lung-PTV, spinal cord, oesophagus, heart, trachea, brachial plexus and chest wall were outlined as organs at risk (OAR) on CT simulation.

### Dose prescription and treatment planning

For each patient, two RapidArc plans (Varian Medical System®) were calculated by the same radiophysicist using the Eclipse treatment planning software (Helios, Varian Medical System®) with the Analytic Anisotropic Algorithm v.10. For the first plan, the objective was to deliver 48 Gy in PTV_CT_ in 4 fractions of 12 Gy. For the second plan, the objective was to deliver 48 Gy in PTV_CT_ and to guide hotspots in BTV with SIB. Both plans adopted the following ROSEL recommendations: 95% of the PTV_CT_ had to receive at least 100% of the prescribed dose and 99% of the PTV_CT_ at least 90% of the prescribed dose. The maximum dose within the PTV_CT_ (Dmax PTV_CT_) had to be lower than 125% of the prescribed dose. Additional constraints included the D2cm (maximal dose at 2 cm of the PTV_CT_), the R50% and R100% (ratios between PTV_CT_ and the 50% and 100% isodoses, respectively) allowing to verify the rapid fall-off of the dose outside the PTV_CT_. The OARs dose constraints were in accordance with the RTOG 0915 trial. The Dmax of the spinal cord, the brachial plexus, the oesophagus, the heart and the trachea were inferior to 26, 27.2, 30, 34 and 34.8 Gy, respectively. For the chest wall, the dose to one cm^3^ (D1cc) was kept below 32Gy.

RapidArc plans were performed with three 180° coplanar arcs using 6MV photons at a maximum dose rate of 600 monitor units (MUs) per minute. Collimator angles of 15, 30 and 330° were used to minimize the tongue-and-groove effect. For the first plan, 12 Gy per fraction were prescribed at the barycentre of the PTV_CT_. For the second plan, 12 Gy and 13.8 Gy (115% of the prescribed dose) were prescribed with SIB in PTV_CT_ and BTV, respectively.

### Dosimetric analysis

In order to assess hotspots, we calculated the volume of the 115% isodose (V_iso115%_) and the Dice Similarity Coefficient (DSC). DSC allows to verify the location of the 115% isodose, and was defined as follows: DSC = (V_iso115%_∩V_BTV_)/(V_iso115%_∪V_BTV_). Ideally, DSC is equal to 1.

## Results

The mean PTV_CT_ and BTV were 36.7 (±12.5) and 6.5 (±2.2) cm^3^, respectively. For both plans, ROSEL recommendations were respected. The SIB technique (plan 2) led to similar target coverage than that obtained with conventional prescription on PTV_CT_ (plan 1): the mean D95%, D99% and the Dmax of the PTV_CT_ were 103.7% ± 1.3, 101.6% ± 2.1 and 123.5% ± 1.9 without SIB, versus 103.7% ± 2.4, 101.3% ± 2.1 and 124.4% ± 0.3 with SIB. The rapid fall-off of the dose outside the PTV_CT_ was equivalent for both plans: the mean D2cm, R50% and R100% were 62.4% ± 3.9, 4.6% ± 0.7 and 1.1% ± 0.08 without SIB, versus 66.2% ± 3.9, 4.7% ± 0.9 and 1.09% ± 0.1 with SIB.

For both plans, constraints of doses to the OARs were respected, except for the dose to the chest wall for patient 1, who presented a tumour attached to the rib.

**Figure 1:**
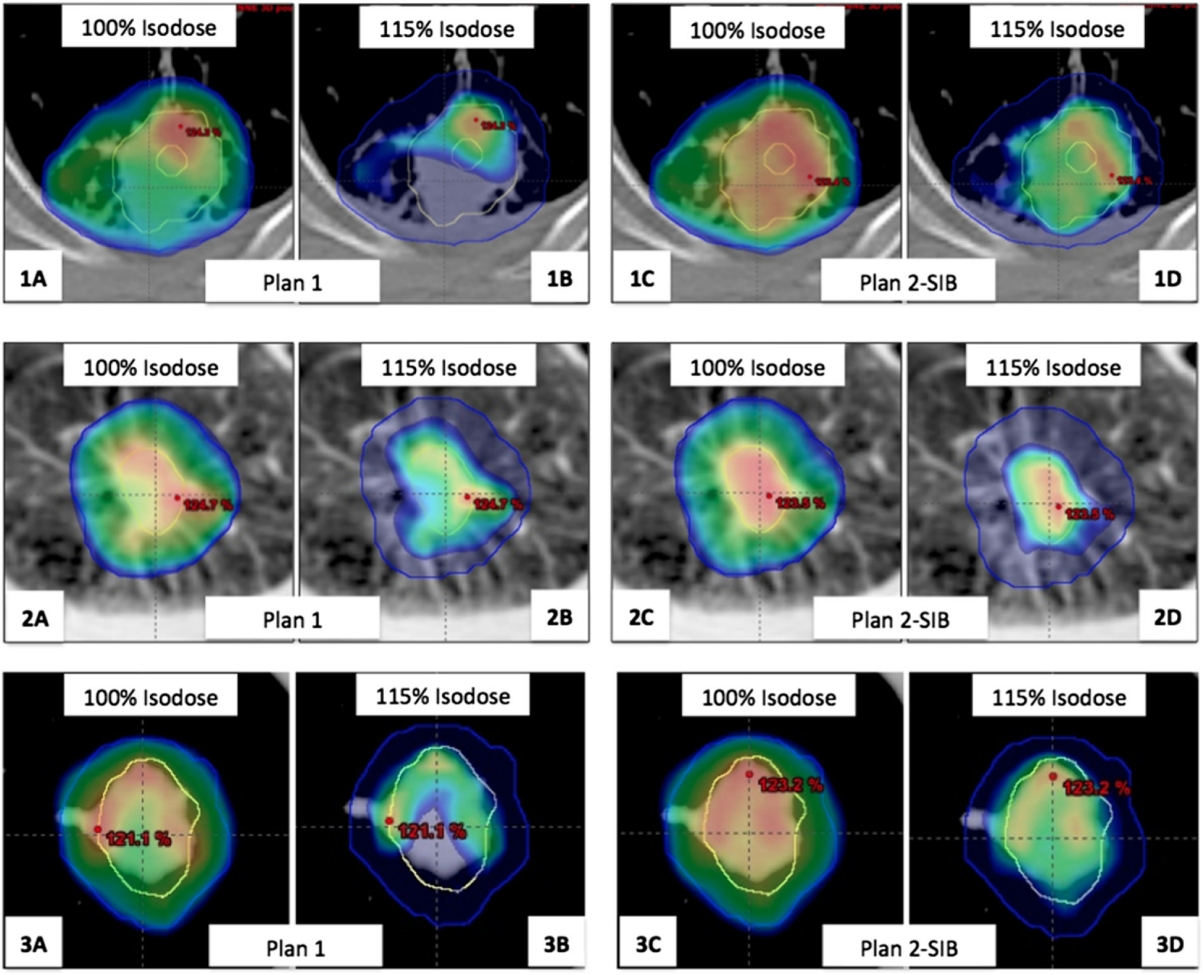
**Comparison of both plans for the three patients: plans 1 in (A) and (B) and plans 2 with SIB in (C) and (D).** PTV_CT_ is shown in blue and BTV in yellow. With both techniques, the target coverage of the PTV_CT_ with the 100% isodose is adequate but the 115% isodose is more focused on the BTV with the SIB technique.

To assess the ability of the SIB to guide hotspots, we evaluated the volume of the 115% isodose and the location of this isodose through the analysis of the DSC (Table 1). The Viso115% was more important in plan 2, with a 33% and 16% increase for patients 1 and 3, respectively. On the contrary, for patient 2 the Viso115% was bigger in the plan 1 with a difference of 85%. Analysis on the location of the 115% isodose revealed that SIB technique improves DSC with a mean of 0.31 for plan 1 and 0.45 for plan 2.. SIB technique’s ability to guide hotspots in the BTV and to decrease arbitrary hotspots is illustrated in Figure 1.

**Table 1:** **The 115% isodose and the dice similarity coefficient (DSC)**

**Patient**	**Viso115% (cm** ^**3**^ **)**		**DSC BTV-Viso115% (%)**	
	**Plan 1**	**Plan 2-SIB**	**Plan 1**	**Plan 2-SIB**
**1**	14	18.6	0.26	0.45
**2**	22.2	12	0.21	0.4
**3**	10	11.6	0.45	0.51
**Mean (± SD)**	15.4 ± 6.2	14.1 ± 3.9	0.31 ± 0.13	0.45 ± 0.06

## Discussion

One limitation of this study is the lack of 4D-CT simulation, which impedes a good estimation of the ITV. An isotropic margin was also added automatically around the GTV_CT_, probably leading to an overestimation of the PTV_CT_. The present investigation, however, does not focus on target volume definition, but rather on the use of PET and VMAT to control and guide hotspots in SBRT. In this respect, the study demonstrates that it is feasible to guide dose prescription heterogeneity on FDG-PET data with VMAT and SIB. Indeed, plans 1 and 2 respected conventional dosimetric criteria of SBRT equally (D95% PTV_CT_, 99% PTV_CT_, Dmax PTV_CT_, D2cm, R50% and R100%) and imparted equivalent doses to the OARs. In contrast, better DSC was computed with plan 2. In this study, we chose to perform a SIB with a dose of 115% of the prescribed dose, because the BTV defined on 4D-FDG-PET is relatively large considering the ITV. FDG-PET was used because its ability to detect sites of relapse after radiotherapy is recognized. After conventional fractionated radiotherapy treatments, relapses are often located inside the area of highest uptake on baseline FDG-PET scan [7,8]. That’s why some authors try to increase the radiation dose inside this BTV. Our purpose was based on this idea and was to integrate data of FDG-PET in SBRT focusing hotspots on BTV. Other PET tracers like hypoxia or proliferation tracers could be of interest to individualize more specific BTVs. These BTVs issued from more specific tracers are often smaller than those defined with FDG. The choice of isodose (115, 120% or higher) to focus hotspots could also vary depending on the tracer and volume of BTV. Whatever the choice of the tracer, an important difficulty stays the choice of the method of PET images segmentation. Many automatic segmentation methods are described in the literature and in the absence of a confrontation with gold standards or surgical specimens, it’s impossible to promote one method. More complex segmentation methods as gradient based or stochastic methods seem more robust than thresholding methods based on SUV [9,10].

VMAT is particularly interesting to plan and deliver SBRT. The number of studies concerned with SBRT and VMAT has incredibly increased these last years. Several dosimetric comparisons confirmed an important reduction in treatment time compared to other delivery techniques like three-dimensional conformal radiotherapy, Cyberknife or Tomotherapy [6,11-14] as well as a reduction of patient movements caused by discomfort and a reduction of the consecutive intra-fraction motion. VMAT can achieve better conformity, sharper dose fall-off outside the PTV and lower dose to normal lung [12-15].

However, concerns about use of modulation therapy for SBRT for tumors subject to respiratory motion have been previously highlighted with the “interplay effect” [16]. This effect describes the interplay between MLC motion and tumour motion with risk of hotspots or cold spots inside the tumor. Recent series have investigated the impact of tumour motion on 4D dose calculation for lung SBRT and found a negligible interplay effect, with differences between 3D and 4D calculations around 1% [17,18]. Several reasons may explain this negligible effect. First, since high doses per fraction are delivered during SBRT, most VMAT segments receive a large number of MUs with a long segment delivery time (on the order of the breathing period). Moreover, modulation for lung SBRT is low with few OARs. Also, most segments contain an aperture shape conformed to the PTV and few segments have leaves blocking a part of the tumor. In the same way, the gantry speed is in general low to be able to deliver a large number of MUs at each segment. Few clinical studies about modulation intensity for lung SBRT are published but results of the Mayo clinic with IMRT [19], of Yamashita et al. [20] with VMAT or Navarria et al. [15] with VMAT unflattened beams (FFF) are good with excellent local control.

## Conclusion

Use of PET to guide dose prescription heterogeneity with VMAT and SIB for lung cancer is feasible and seems promising. These preliminary results have to be confirmed with more patients and confronted to a clinical study. Moreover, other PET tracers more specific than FDG could be evaluated for this dose painting.
